# Erlotinib in a patient with vaginal carcinoma and pulmonary metastasis: A case report

**DOI:** 10.3892/ol.2014.1805

**Published:** 2014-01-15

**Authors:** QING MA, YAO-YAO REN, XIA LIU, TING GONG, DIAN-SHENG ZHONG

**Affiliations:** Department of Oncology, General Hospital of Tianjin Medical University, Tianjin 300052, P.R. China

**Keywords:** erlotinib, vaginal carcinoma, pulmonary metastasis

## Abstract

Erlotinib is an epidermal growth factor receptor tyrosine kinase inhibitor. It is widely used in the treatment of advanced non-small cell lung cancer and pancreatic cancer. However, there are currently no reports of the efficacy of erlotinib in patients with metastatic vaginal carcinoma. A 48-year-old female with vaginal carcinoma was diagnosed with lung metastasis four years following surgery. The patient received three cycles of chemotherapy but could not tolerate further treatment due to the side effects. Next, erlotinib was administered, prompting a partial response and disease stabilization for 9 months prior to disease progression. While the main treatments for vaginal carcinoma with distant metastasis are chemotherapy and radiotherapy, this case supplies preliminary evidence that erlotinib may have activity in these patients. Further studies are required to determine the potential of this therapy.

## Introduction

Vaginal neoplasms account for ~2% of all female genital neoplasms (1) and are among the rarest malignancies. Thus, there is little information in the literature concerning this type of cancer, particularly with regard to therapy (2). Since the etiology of vaginal carcinoma is similar to that of carcinoma of the cervix, patients with advanced vaginal cancer can be treated with the standard cervical cancer regimen of cisplatin-based chemotherapy. If chemotherapy lacks efficacy or the patient is in poor physical condition, then determining the best treatment strategy can be difficult. Erlotinib is an epidermal growth factor receptor (EGFR) tyrosine kinase inhibitor (TKI). This drug is widely used in the treatment of advanced non-small cell lung cancer (NSCLC) and pancreatic cancer, primarily to disrupt the EGFR signaling pathway. EGFR is also expressed in cervical cancer and serves as a strong prognostic indicator (3). Previous studies have also shown that TKIs may be suitable for use as second or third lines of therapy for cervical cancer (4). The current report presents the case of a patient with metastatic vaginal carcinoma who was administered erlotinib and remained stable for 9 months following the failure of first-line chemotherapy.

## Case report

**Figure 1:**
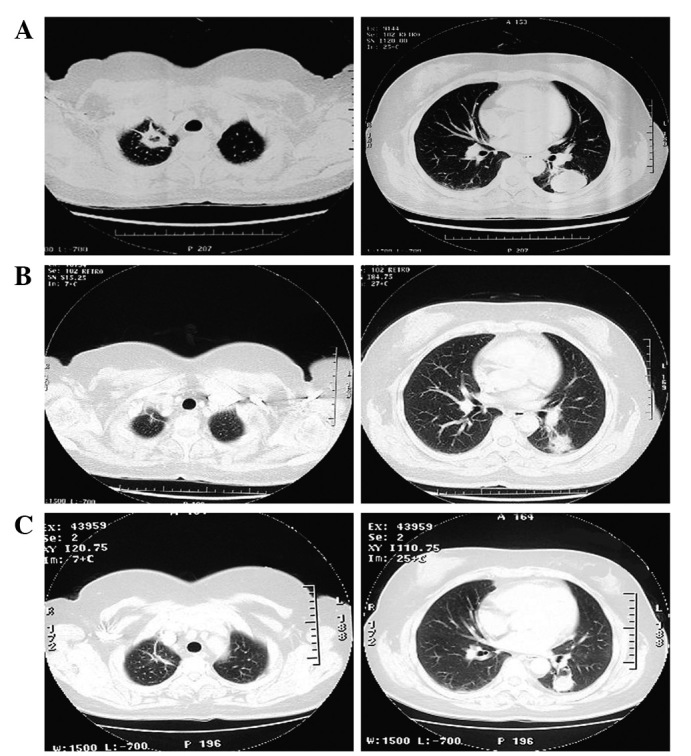
Computed tomography radiographs showing changes in pulmonary metastasis in response to erlotinib therapy. Axial and coronal views of a (A) baseline scan prior to starting erlotinib, (B) partial response after one month of erlotinib treatment and (C) stable disease after three months.

A 48-year-old female was diagnosed with vaginal carcinoma in 2009. The patient underwent 15 rounds of radiation therapy prior to surgery and an additional 15 rounds following surgery. Later, the patient underwent 6 cycles of chemotherapy with paclitaxel plus carboplatin. During this period, the patient experienced grade 2 bone marrow suppression on two separate occasions. After 6 cycles, imaging indicated remission. The patient underwent routine disease surveillance, but developed hemoptysis without fever or chest pain 1 year after diagnosis. Chest computed tomography (CT) revealed multiple nodules in the lungs. A pair of dominant soft tissue masses measuring 5 and 3 cm were located in the left lower lung and right upper lung, respectively. Mediastinal lymph nodes were enlarged. Carcinoembryonic antigen levels were elevated to 35.9 mg/dl. Following percutaneous lung biopsy, pathological examination confirmed a diagnosis of squamous cell carcinoma associated with the vaginal carcinoma. The patient went on to receive chemotherapy with paclitaxel (135 mg/m^2^ on day 1) plus carboplatin (area under the curve, 5 × 500 mg on day 1) every 21 days. After 3 cycles, the lesions became stable (Fig. 1), but the patient began to feel nauseous and fatigued from fourth-degree bone marrow depression, preventing tolerance of further treatment. The patient refused second-line chemotherapy. After being fully informed about the uses of erlotinib and the lack of evidence for the efficacy of the drug in this setting, the patient was orally administered 150 mg/day erlotinib. Following six weeks of erlotinib therapy, the target lesions were assessed and found to have reduced in size by 40–50%. This was consistent with a partial response. The target lesions continued to decrease in size, and stabilized three months following therapy (CT showed maximum diameters of 2.8 and 1.5 cm, respectively). The patient’s condition remained stable for a total of nine months. Subsequently, the patient decided to terminate therapy with the exception of palliative care. Progressive disease was evident in the lungs 16 months later, and eventually the patient succumbed to respiratory failure. This study was approved by the Ethics Committee of Tianjin Medical University (Tianjin, China). The patient provided written informed consent.

## Discussion

Primary vaginal cancer is a rare condition accounting for 1–3% of all gynecological malignancies. While the major relapse pattern for vaginal carcinoma is local recurrence, the development of lesions outside the pelvis has also been reported (5,6). The patient of the present case report had metastatic lesions in the lungs. Since the patient could not tolerate additional courses of carboplatin and paclitaxel and refused second-line chemotherapy, erlotinib was administered in an attempt to control the disease.

Erlotinib is the standard therapy for advanced NSCLC with EGFR mutation and has also been approved for the treatment of unselected chemorefractory advanced NSCLC and for maintenance therapy following first-line chemotherapy (7). While it is known that EGFR-TKIs, including erlotinib, mainly benefit EGFR-mutated adenocarcinomas, a pooled analysis was conducted that demonstrated an efficacy of gefitinib for non-adenocarcinoma NSCLC patients harboring the EGFR mutation (8). A phase III trial (BR.21) evaluated the effects of erlotinib in patients who had been treated for NSCLC. Among the participants, 30% had squamous cell histology. Patients with wild-type EGFR showed some survival benefit when treated with erlotinib alone (9). Subgroup data from the global, multicenter, randomized, double-blind, placebo-controlled study, SATURN, showed that erlotinib can be beneficial regardless of patient age, race, histology or smoking history. Squamous cell carcinoma patients, who had not previously been considered suitable for EGFR-TKI treatment, showed some benefit from treatment with EGFR-TKIs. Patients who received erlotinib maintenance therapy following chemotherapy had a 24% lower risk of disease progression. Patients with wild-type EGFR had a 22% lower risk of progression (10).

The use of EGFR-TKIs outside of lung and pancreatic cancer is uncommon. A multicenter, open-label, non-comparative, phase II trial was previously performed to evaluate the clinical outcomes of gefitinib in cervical cancer in 28 patients. The condition of 6 patients (20%) was stabilized for a median period of 111.5 days. The median time to progression was 37 days and the median overall survival time was 107 days. Disease control did not appear to be correlated with levels of EGFR expression. Gefitinib was well tolerated. The use of gefitinib (or other EGFR-TKI therapies) in cases of recurrent disease resistant to standard treatment may warrant further investigation (4).

To the best of our knowledge, the present study is the first report of the use of erlotinib in a patient with vaginal cancer with metastatic disease in the lungs. Although the evidence is somewhat subjective, the results are promising. Further investigation of the use of this class of drugs in this setting is warranted.
